# Supplementary material for: Herbal Teas and Drinks: Folk Medicine of the Manoor Valley, Lesser Himalaya, Pakistan
Source: Plants (Basel). 2019 Dec 7;8(12):581. doi: 10.3390/plants8120581 (PMC6963793; doi:10.3390/plants8120581)
Supplement: Supplementary file 1 [file plants-08-00581-s001.pdf]

plants MDPI

Table 1. Comparison of the present study with previous studies at regional, neighboring and global level.

Study Area	Number of recorded Plants Species of Aligned Areas	Total Species Common in Both Area	Species Enlisted Only in Aligned Areas	Species Enlisted Only in Our Study Area	Common Species with Similar Uses	Common Species with Dissimilar Uses	Sorensen's Similarity Index (QS%)	Rahman's Similarity Index (RSI%)	Citation
Kaghan Valley, Pakistan	30	4	26	23	2	2	**16.00**	**4.00**	Jamal et al. [52]
Naran Valley, Pakistan	101	10	91	17	2	8	**18.35**	**1.83**	Khan et al. [42]
Abbottabad, Northern Pakistan	47	8	39	19	4	4	**27.12**	**6.78**	Qureshi et al. [28]
Kaghan Valley, Pakistan	75	3	72	24	1	2	**6.19**	**1.03**	Jan et al. [50]
Shogran Valley, Pakistan	107	11	96	16	2	9	**19.47**	**1.77**	Matin et al. [41]
Shogran Valley, Pakistan	50	2	48	25	1	1	**5.41**	**1.35**	Ume-Ummara et al. [53]
Himalaya, Pakistan	89	9	80	18	2	7	**18.18**	**2.02**	Abbasi et al. [51]
Kotli, Azad Jammu Kashmir, Pakistan	112	2	110	25	0	2	**2.94**	**0.00**	Ahmad et al. [46]
Kotli, Azad Jammu Kashmir, Pakistan	50	2	48	25	0	2	**5.41**	**0.00**	Ajaib and Khan [47]
Siran Valley, (Mansehra), Pakistan	143	0	143	27	0	0	**0.00**	**0.00**	Ahmad et al. [43]
Nathiagali, Pakistan	31	1	30	26	0	1	**3.51**	**0.00**	Tariq et al. [49]
Swat, North Pakistan	106	14	92	13	3	11	**26.42**	**2.83**	Akhtar et al. [29]
Azad Jammu and Kashmir, Pakistan	73	4	69	23	0	4	**8.60**	**0.00**	Rashid et al. [23]

Sarban Hills, Abbottabad, Pakistan	74	10	64	17	5	5	**24.39**	**6.10**	Ijaz et al. [5]
Deosai Plateau, Gilgit Baltistan, Pakistan	50	0	50	27	0	0	**0.00**	**0.00**	Bano et al. [44]
Thakt-e-Sulaiman Hills, Pakistan	51	3	48	24	0	3	**8.22**	**0.00**	Ahmad and Pieroni [48]
Lakki Marwat, Pakistan	72	5	67	22	0	5	**11.11**	**0.00**	Ullah et al. [31]
Chungtia village, Nagaland, India	135	3	132	24	0	3	**5.16**	**0.00**	Kichu et al. [13]
Aladaglar, Nigde-Turkey	110	0	110	27	0	0	**0.00**	**0.00**	Ozdemir and Alpinar [45]
Thanchi, Bandarban Hill Tracts, Bangladesh	84	3	81	24	1	2	**5.66**	**0.94**	Kadir et al. [30]

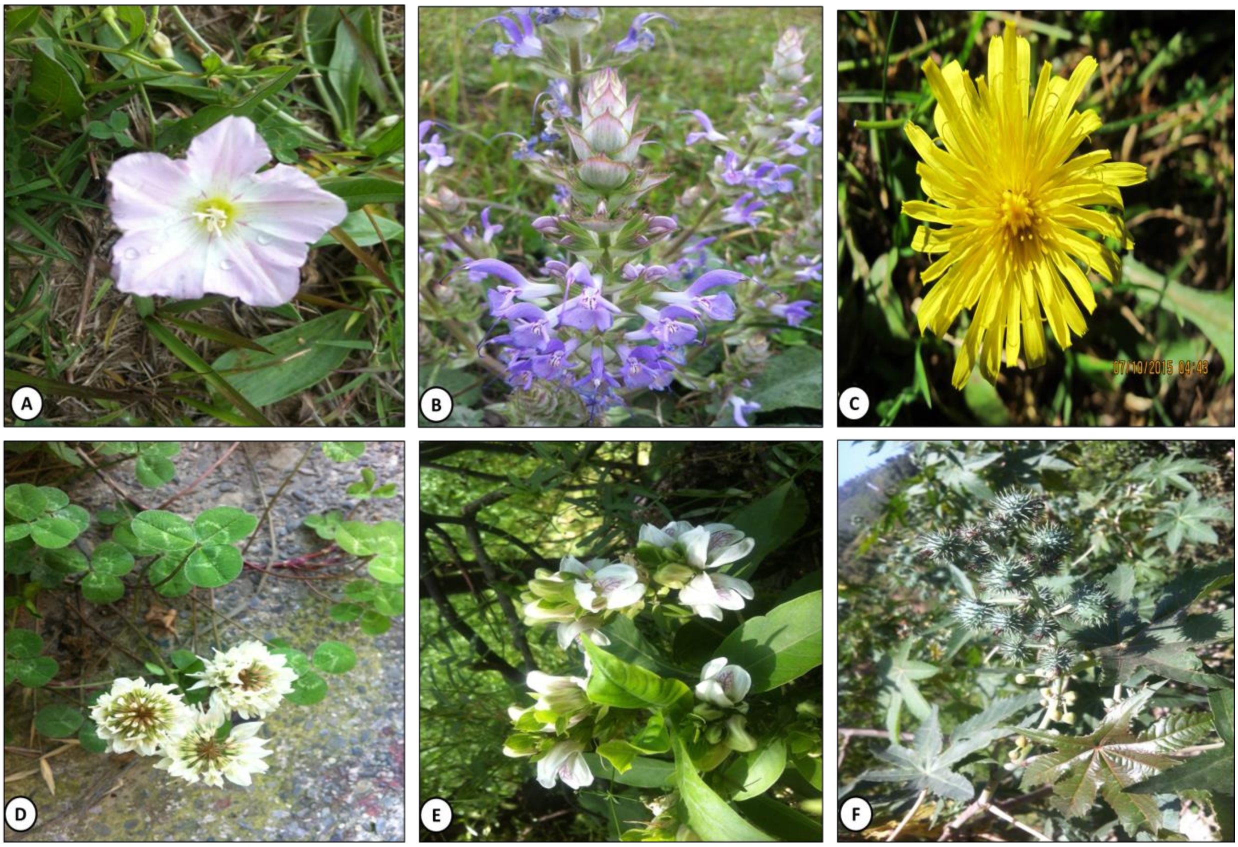

Figure S1. Some medicinal plants of the study area. (A) Convolvulus arvensis**, (B)** Salvia moorcroftiana**, (C)** Taraxacum officinale**, (D)** Trifolium repens**, (E)** Justicia adhatoda**, (F)** Ricinus communis**.**
